# *Xanthium strumarium* L. Extracts Produce DNA Damage Mediated by Cytotoxicity in *In Vitro* Assays but Does Not Induce Micronucleus in Mice

**DOI:** 10.1155/2014/575197

**Published:** 2014-06-15

**Authors:** Janet Piloto Ferrer, Renata Cozzi, Tommaso Cornetta, Pasquale Stano, Mario Fiore, Francesca Degrassi, Rosella De Salvia, Antonia Remigio, Marbelis Francisco, Olga Quiñones, Dayana Valdivia, Maria L. González, Carlos Pérez, Angel Sánchez-Lamar

**Affiliations:** ^1^Departamento de Genotoxicidad, Centro de Investigación y Desarrollo de Medicamentos (CIDEM), Avenida 26, No. 1605 e/ Puentes Grandes y Boyeros, La Habana, Cuba; ^2^Dipartimento di Scienze, Università degli Studi “Roma TRE”, Via G. Marconi 446, 00146 Roma, Italy; ^3^Istituto di Biologia e Patologia Molecolare, CNR, Via degli Apuli. 4, 00185 Roma, Italy; ^4^Departamento de Bioquímica, Instituto de Ciencias Básicas y Preclínicas “Victoria de Girón” (ICBP-UCMH), Calle 146, No. 3102 Esq 31, Playa, La Habana, Cuba; ^5^Departamento de Biología Vegetal, Laboratorio de Toxicología Genética, Facultad de Biología, Universidad de la Habana, Calle 25, No. 455, e/ I y J, Vedado, C. Habana, Cuba

## Abstract

*Xanthium strumarium* L. is a member of the Asteraceae commonly used in Cuba, mainly as diuretic. Some toxic properties of this plant have also been reported and, to date, very little is known about its genotoxic properties. The present work aims was to evaluate the potential cytotoxic and genotoxic risk of whole extract from *Xanthium strumarium* L. whole extract of aerial parts. No positive response was observed in a battery of four *Salmonella typhimurium* strains, when exposed to concentrations up to 5 mg/plate, with and without mammalian metabolic activation (liver microsomal S9 fraction from Wistar rats). In CHO cells, high concentrations (25–100 *μ*g/mL) revealed significant reduction in cell viability. Results from sister chromatid exchanges, chromosome aberrations, and comet assay showed that *X. strumarium* extract is genotoxic at the highest concentration used, when clear cytotoxic effects were also observed. On the contrary, no increase in micronuclei frequency in bone marrow cells was observed when the extract was orally administered to mice (100, 500, and 2000 mg/Kg doses). The data presented here constitute the most complete study on the genotoxic potential of *X. strumarium* L. and show that the extract can induce *in vitro* DNA damage at cytotoxic concentrations.

## 1. Introduction

*Xanthium strumarium* L. (Asteraceae), a medicinal plant commonly found as a weed, is widely distributed in North America, Brazil, China, Malaysia, and hotter parts of India. The herb is traditionally used mostly in treating several aliments. In Cuba,* X*.* strumarium* is commonly called “guisazo de caballo” and it has been used as diuretic [1]. The plant has been employed for a long time in folk therapy. More than 20 properties have been attributed to the decoctions and tinctures from the leaves and roots, for example, antirheumatic, antisyphilitic, appetiser, diaphoretic, diuretic, emollient, laxative, and sedative activities. Other actions have been confirmed by experimental pharmacology: anti-inflammatory, analgesic [2, 3], antibacterial, anticancer [4, 5], antifungal [6], antihypoglycemic [7], antimitotic [8, 9], antitrypanosomal, antimalarial [10], and diuretic activities [11].

The toxicity reported for the plant suggests that potential negative effects on human health elicited by the consumption of herbal remedies based on* X. strumarium* should be considered. Genotoxicity is one of the most important toxic effects and its relevance is greater as we are dealing with events taking place at sublethal doses leading to long-term effects such as cancer and reproductive diseases.

The present work aims at assessing the potential cytotoxic and genotoxic risk of such extract. For this purpose, different genetic endpoints were assayed, in order to evaluate DNA damage at different genetic expression levels. We assessed the cell viability of* Xanthium strumarium* L. extracts on Chinese hamster ovary (CHO) cells using MTT assay. Then we tested the mutagenicity of the extract in four short-term assays:* Salmonella*/microsome (the Ames test) for gene mutation, cytogenetic assays on CHO cells for chromosomal damage* in vitro*, comet assay for primary DNA damage, and the micronucleus test in mouse bone marrow for clastogenic and aneugenic effects* in vivo*.

## 2. Materials and Methods

### 2.1. Plant Material and Extract Preparation

*Xanthium strumarium* L. was collected from the Medicinal Plants Experimental Station “Dr. Juan Tomás Roig” in Güira de Melena, Artemisa. A voucher specimen with number ROIG 4594 was deposited at the herbarium of this institution. A fluid extract was prepared from the dried material by hydroalcoholic extraction in four rounds of percolation. It was concentred under reduced pressure to obtain a raw extract (whole extract), kept in sealed containers at 4°C. The chemical characterization of the extract was performed by qualitative analysis as described by Miranda and Cuellar [12]. This analysis revealed a higher polysaccharides content, phenol, saponin, terpenes, sesquiterpene lactones, steroids, cumarin, and flavonoids.

### 2.2. Negative Control

In all cases (*in vivo *and* in vitro *assays) the negative control was the appropriate solvent used to dissolve plant extract and all the employed substances. Dimethyl sulfoxide (DMSO) was the solvent in* in vitro* assays and ethanol in* in vivo* test.

### 2.3. Positive Control

The following positive controls were used: aminofluorene (2AF), sodium azide, cyclophosphamide (CP), 9-aminoacridine (9AA), and pichrolonic acid (PA) in Ames test; CP in mouse bone marrow micronucleus; mitomycin C and X-ray in chromosome aberrations assays; hydrogen peroxide (H_2_O_2_) in the alkaline comet assay in CHO cells.

### 2.4. *In Vitro* Assays

#### 2.4.1. Salmonella/Microsome Assay

The plate-incorporation mutagenicity assay was performed as described by Maron and Ames [13] using* Salmonella typhimurium *tester strains TA 1535, TA 1537, TA98, and TA100. Five concentrations of the extract were tested in triplicate plates in a single experiment, covering a range from 50 to 5000 *μ*g of solids/plate. Liver microsomal S9 fraction was prepared from Wistar rats treated with phenobarbital and 5.6 benzoflavone. S9 mix used for exogenous metabolic activation contained a 4% S9 fraction in a solution of cofactors (NADP-regenerating system). Revertant colonies were counted manually after 72 h of incubation at 37°C.

#### 2.4.2. Cell Culture and Cytogenetic Assays in Chinese Hamster Ovary (CHO) Cells

CHO cells were grown in Ham F10 medium supplemented with 10% foetal bovine serum, 100 IU/mL penicillin, 100 IU/mL streptomycin, and 2 mM L-glutamine in a 5% CO_2_ humidified atmosphere at 37°C. All experiments were performed, seeding 4 × 10^5^ cells per 25 cm^2^ flask. Under these conditions, the cell cycle of this line was approximately 12 h. 

*(1) MTT Cytotoxicity Assay*. The MTT [3-(4, 5-dimethylthiazol-2-yl)-2, 5-diphenyltetrazoliumbromide] cytotoxicity assay was carried out in accordance with the protocol described by Mosmann [14] with some modifications. Added to each well of a culture microplate were approximately 1 × 10^4^ CHO cells. The cells were exposed to extract for 24 h. At the end of this period, the cells were incubated with MTT (5 mg/mL) for 4 h. The plates were read in a microplate spectrophotometer (OMEGA) at 550 nm. The IC_50_ were determined from a dose-response curve by using 5 different concentrations (6.25, 12.5, 25, 50, and 100 *μ*g/mL). Analyses were done in triplicate for each concentration.

*(2) Sister Chromatid Exchanges (SCE) Assay*. Cells were treated for 3 h with a recovery time of 24 h or were continuously exposed for 27 h to* X. strumarium* extracts. Cultures received BrdUrd at the final concentration of 1.5 *μ*g/mL during the last 24 h of incubation. 5 × 10^−7^ M colchicine was always added 2 h before fixation. As previously described, FPG Hoechst- Giemsa technique modified by De Salvia et al. [15] was used for differential staining of sister chromatids. The scoring of SCE was according to Sánchez-Lamar et al. [16] and 20 metaphases per experimental point were analyzed. 

*(3) Chromosome Aberration (CA) Assay*. The chromosome aberration test was conducted according to the international guidelines for this assay [17]. Exponentially growing CHO cells were treated according to the following experimental schedule. Two types of treatment were employed: 3 h treatment with 15 h recovery time and 18 h continuous treatment until harvesting. 5 × 10^−7^ M colchicine was added 2 h before harvesting. After trypsinisation, cells were treated with a hypotonic solution, fixed with acetic acid and methanol (1 : 3) solution, and stained as reported by Galloway et al. [18]. At least 150 metaphases were scored for each dose of plant extract. 

*(4) Alkaline Comet Assay*. The alkaline comet assay was performed as described by Cornetta et al. [19] with minor modifications. Exponentially growing CHO cells were treated for 3 h with different concentrations (5, 15, and 45 *μ*g/mL) of the extract. At the end of treatment 40 *μ*L of cellular suspension was mixed with 5 mL trypan blue for 3 min. Cell suspension was pipetted onto a glass microscope slide and analyzed under a phase-contrast microscope, for calculating the proportion of nonstained (viable) cells. Immediately after treatment 20 *μ*L of cells was mixed with 180 *μ*L of 0.7% low melting point agarose in PBS (Ca and Mg free) at 37°C and immediately pipetted onto a frosted glass microscope slide precoated with a layer of 1% normal melting point agarose, similarly prepared in PBS. Two slides were prepared for each experimental point. The agarose was allowed to set at +4°C for the necessary time and the slides incubated in a lysis solution (2.5 M NaCl, CAS number 7647-14-5, 10 mMTris-HCl,CAS number 77-86-1, 100 mM Na_2_EDTA, CAS number 6381-92-6, NaOH, CAS number 1310-73-2, to pH 10, 1% Triton, CAS number 9002-93-1, and 10% DMSO, CAS number 67-68-5) for 50 min. After lysis, slides were placed onto a horizontal electrophoresis unit containing fresh buffer (1 mM Na_2_EDTA, 300 mM NaOH, pH 13) for 20 min to allow for DNA unwinding. Electrophoresis was conducted for 15 min (25 V, 300 mA) at 4°C. Subsequently, slides were gently washed in neutral buffer solution for 5 min (0.4 MTris-HCl, pH 7.5), fixed in 100% freshly methanol for 3 min, and stained with ethidium bromide (2 *μ*g/mL, CAS number 001239458). Slides were analyzed using a fluorescence microscope (Leica) equipped with a camera. Fifty comets on each slide, coded and blindly scored, were acquired using “I.A.S.” software automatic image analysis system purchased from Delta System (Rome, Italy). To quantify the induced DNA damage the Tail DNA (TD), which is a measure of the percentage of migrated DNA in the tail, was used [20].

### 2.5. *In Vivo* Assays

Swiss mice obtained from the National Centre for Production of Laboratory Animals (CENPALAB, Cuba) were bred in our laboratory animal-care facility and employed in the present work. Adult healthy animals, 8–10 weeks old, weighing 25–30 g of either sex were used. Each experimental group consisted of five animals. All the animal studies reported in this work were carried out in accordance with the Cuban regulations on the protection of animals (Código Práctico para el uso de Animales de Laboratorio, Centro Nacional para la Producción de Animales de Laboratorio, CENPALAB) and the Declaration of Helsinki. All experimental protocols were revised by the Animal Care and Use Committee of the Faculty of Biology, University of Havana, and conducted humanely.

#### 2.5.1. Mouse Bone Marrow Micronucleus (MN) Assay

The extract was administered p.o. in a volume of 10 mL/kg. Three dose groups (500, 1000, and 2000 mg/kg b.w) were assayed in both sexes. The usual subacute protocol of two administrations 24 h apart and a single sacrifice 24 h later was employed [21]. Mouse bone marrow was obtained from five animals for each dose of extract and controls as reported by Schmid [22]. Smears were stained with May-Grunwald and Giemsa (Merck) and analysed for the presence of MN. Genotoxicity index (GI), which is the percentage of polychromatic erythrocytes (PCE) with micronuclei (PCE-MN), was calculated from 2000 cells and the cytotoxicity index (CI) was determined as the ratio of PCE to normochromatic erythrocytes (NCE) (PCE/NCE) calculated from 250 erythrocytes perslide.

### 2.6. Statistical Analysis

Means and standard errors were determined using Kolmogorov-Smirnov test. Controls and treated samples were compared using the Student's* t*-test, the two-way ANOVA, the bi- and trifactorial ANOVA, the Dunnett's test, Fisher test, and Mann-Whitney *U* test, depending on the assay [23]. In* in vitro* cytogenetic assays, the tested concentrations of extract were considered positive when statistically significant increases in total alterations frequency (CA or SCE) exceeded the historical control mean values. In* Salmonella* test, the controls and treated samples were compared using SALANAL (Salmonella Assay Analysis, version 1 US Environmental Protection Agency).

## 3. Results

### 3.1. Salmonella/Microsome Assay

Reversion frequencies observed in the* Salmonella*/microsome mutagenicity test are presented in Tables 1 and 2. No increase of revertants per plate was observed in any of the four standard bacterial strains used in the assay, with and without extrinsic metabolic activation provided by S9 mix, in the relatively wide range of concentrations tested (50–5000 *μ*g/plate).

### 3.2. MTT Cytotoxicity Assay

**Figure 1:**
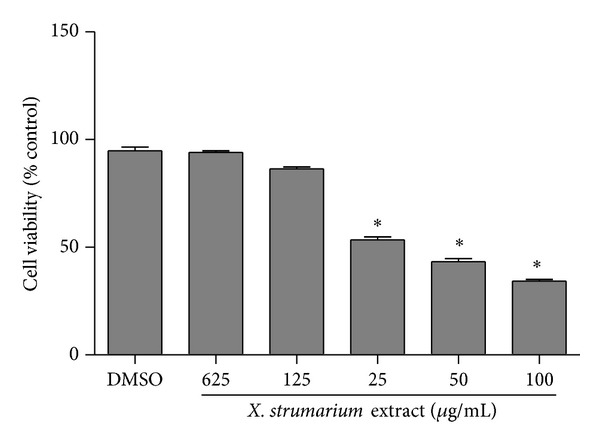
Cytotoxic effects of* X. Strumarium* extract in CHO cells by the MTT assay. Cells were exposed for 24 h to different concentrations (6.25, 12.5, 25, 50, and 100 *μ*g/mL) of the extract. Negative control: DMSO (1%). Each value represents the mean ± SE of three independent experiments. Statistically significant difference compared to negative control (∗*P* < 0.05, ANOVA and Dunnett's test).

Figure 1 shows the results obtained in the MTT assay in CHO cells treated with* X. strumarium* extract. The readings of the cytotoxicity assay determined spectrophotometrically showed that extract concentrations of 6.25, 12.5, 25, 50, and 100 *μ*g/mL after 24 h of treatment exerted a cytotoxic effect on CHO cells in both dose-time dependent manners. Cytotoxic effect was observed in 25–100 µg/mL. IC_50_ value of* X. strumarium *extract in CHO cells was 20.5 *μ*g/mL

### 3.3. Sister Chromatid Exchanges (SCE) Assay

**Figure 2:**
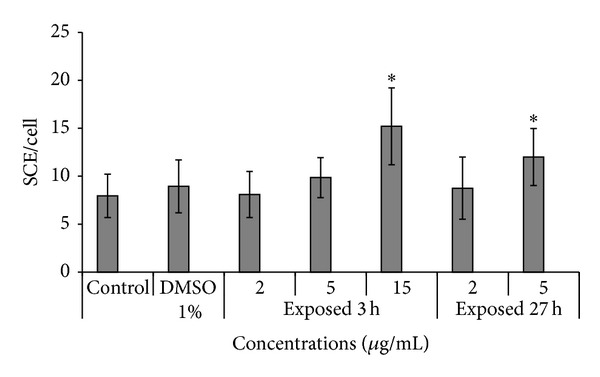
SCE assay analysis in CHO cells treated with crude extract of* X. strumarium* L. (2, 5 and 15 *μ*g/mL). Cells were exposed for 3 h or 27 h to* X. strumarium* extracts. Cultures received BrdUrd at the final concentration of 1.5 *μ*g/mL during the last 24 h of incubation. Negative control: DMSO (1%). Each value represents the mean ± SD of three independent experiments. Statistically significant difference compared to negative control (∗*P* < 0.05, ANOVA and Dunnett's test).

A significant increase of SCE per cell was observed at 15 *μ*g/mL when cells were exposed to the extract for 3 h and then recovered for 24 h in fresh medium (Figure 2). The mean ± SD SCE frequency at 15 *μ*g/mL extract was 15.2 ± 4.01 as compared with 8.95 ± 2.76 in DMSO-treated cells. When a continuous treatment of 27 h was applied, a statistically significant increase in SCE was observed at 5 *μ*g/mL of* X. strumarium* extract. Higher concentrations could not be analysed since a strong antiproliferative effect of the extract was observed and no second mitoses were recorded.

### 3.4. Chromosome Aberration (CA) Assay

The* X. strumarium* extract produced a significant increase in the percentage of CA at the highest tested concentration, that is, 45 *μ*g/mL. At this concentration, the mitotic index was lower than 50% of the mitotic index observed in controls indicating occurrence of cell toxicity from the extract treatment (Table 3). A slight significant increase in the frequency of CA was observed in 25 *μ*g/mL when CHO cells were exposed for 3 h and when cells were continuously treated for 18 h with the* X. strumarium* extract. No mitoses could be observed after 18 h of treatment with 45 *μ*g/mL of extract.

### 3.5. Comet Assay

**Figure 3:**
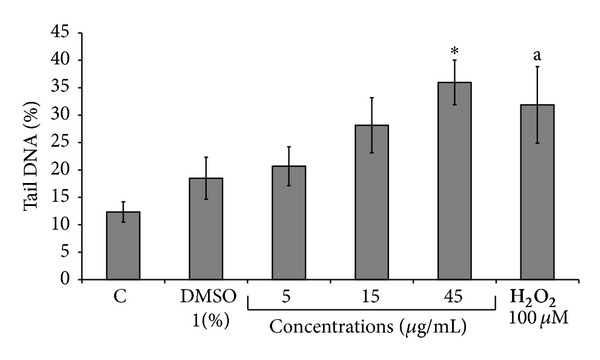
Comet assay analysis in CHO cells treated with fluid extract of* Xanthium strumarium* L. CHO cells were treated for 3 h with different concentrations (5, 15, and 45 *μ*g/mL) of the extract. Positive control: hydrogen peroxide (100 *μ*M for 20 min) and negative control: DMSO (1%). Each value represents the mean ± SD of three independent experiments. Statistically significant difference compared to negative control (*P < 0.05 versus DMSO sample, a *P* < 0.05 versus control at Mann-Whitney *U* test).

The data obtained with comet assay are shown in Figure 3. The percentage of unviable cells never exceeded 20% in each experimental point. A dose-related increase in DNA breaks was observed in treated samples when compared to DMSO-treated one. A significant result (*P* < 0.05) was only obtained at the highest concentration of the extract and hedgehog or apoptotic-like cells were observed.

### 3.6. Mouse Bone Marrow Micronucleus (MN) Assay

The results obtained for mice treated with different doses of* X. strumarium *extract are shown in Table 4. No significant difference in the frequency of MNPCE was observed between mice treated with* X. strumarium *extract and the negative control for both sexes. A high increase in the frequency of MNPCE was detected in mice treated with cyclophosphamide compared to the negative control (*P* < 0.01). No significant differences in the PCE/NCE ratio were observed when comparing mice treated with* X. strumarium *extract and the respective negative control. No signs of toxicity were found in mice at dose levels of 500–2000 mg/kg body weight. There was no mortality in any of the groups; the initial and final weights of the animals were found to be similar to controls.

## 4. Discussion

Plant remedies are traditionally used in Cuba for the treatment of different illness. This knowledge has remarkably been improved through the prioritized policy of the Cuban Ministry of Public Health of developing pharmaceuticals and supplements from natural sources as well as a scientifically sustained phytotherapy. To confirm the pharmacologic properties of* Xanthium strumarium* L., species belonging to the Cuban flora are part of that aim.

*X. strumarium L *is one of the most popular herbal formulas in the world; however, evidence-based information about genotoxicity is limited. Many studies have reported pharmacological efficacies and benefits of* X. strumarium* [2–11], but there is little information on its risk and safety.

Data presented in this report have involved an extensive genotoxic assessment of a complex mixture, the whole extract of* X. strumarium *L. as part of preclinical studies; the use of* in vitro *and* in vivo *assays was decisive in order to obtain a picture of the genotoxic potential of this plant product and to identify potential risk/hazard for human health due to DNA damage and mutation induction.

The species of the genus* Xanthium* are an important source of sesquiterpene lactones which are responsible for most of biological activities attributed to this genus. Sesquiterpene lactones termed xanthanolides, xanthatin, 8-epi xanthatin, and 8-epi tomentosin and others phytocompounds as xanthus-strumarina, hydrochinonas, carboxy-atractyloside, albuminoids and organic acids has been obtained from* X. strumarium* species, which have been reported as cytotoxic substance [2, 4, 5]. Moreover, many reports evidenced that sesquiterpene lactones present in plants have cytotoxic activity [4, 24, 25]. These sesquiterpene lactones trigger mitochondrial membrane transition, loss of mitochondrial membrane potential, and release of proapoptotic mitochondrial proteins leading to caspase activation and apoptotic cell death [26]. Xanthatin also caused a programmed cell death like in trypanosomes as evidenced by a reduction in mitochondrial membrane potential [27].

Our results showed the cytotoxic capacity of* X. strumarium* extract when high concentrations (25–100 *μ*g/mL) for 24 h were applied to CHO cells in* in vitro* experimental conditions. The decrease in cell viability at high concentrations can be related to a toxic effect on the metabolism of the cell or to DNA damage. Coincidentally, precedent studies [2, 4, 5] conducted in* in vitro* experimental conditions reported cytotoxic activity of extracts obtained from this vegetable species. However, acute and subacute administration of root extracts from* X. strumarium* did not produce any toxicity when* in vivo* assay was assessed [28].

The* in vitro *assays used in the present study cover different levels of genotoxic damage expression. In these assays we include toxic concentrations greater than the IC_50_ to confirm cytotoxicity-mediated genotoxicity. A first level was the induction of point mutations at specific* loci *in* Salmonella typhimurium. *The negative outcome of bacterial testing of extract seems to correspond with the absence of potential to induce mutagenic changes in DNA. The second level was the analysis of SCE in mammalian CHO cells. The* X. strumarium *extract produced a significant induction of SCE, which is a sensitive method for the measurement of DNA damage at primary structure level. The* X. strumarium *extract also proved to be clastogenic at the cytogenetic level measuring CA induction in CHO cells only at a concentration that produced severe toxic effects, as assessed by the strong decrease in the mitotic index. The positive result obtained with the highest dose of the extract in the* in vitro* comet assay where hedgehog or apoptotic-like cells were observed indicated that the test substance induces DNA primary damage (both single- and double-strand breaks) in cultured mammalian cells. This result is in line with that obtained with the same concentration in CA analysis.

Maybe the DNA damage can be explained on the basis of a systemic toxic effect. Today, it is known that the double bonds present in the *α*-methylene*γ*-lactone and cyclopentanone moieties confer to sesquiterpene lactones an affinity towards thiol groups of proteins and glutathione inducing oxidative stress in the cell [29]. There is no evidence that chromosomal aberrations induced by sesquiterpene lactones result from direct interaction with DNA or the chromosome structure; rather, an indirect mechanism mediated by cytotoxicity has been suggested [24]. The issue of cell-toxicity-mediated genotoxicity has been intensively addressed and many related endpoints have been proposed [30]. Amongst them, inhibition of key enzymes of energetic metabolism (oxidative phosphorylation) and nucleic acid replication (DNA polymerase) and expression of NFkB and caspase 3, which are proapoptotic and induce DNA damage, have been observed by sesquiterpene lactones [24, 25, 31, 32].

In addition, the present study reveals that the administration of 500–2000 mg/Kg dose of* X. strumarium *extractexhibited no genotoxic activity in our MN* in vivo *assays. There was no mortality or signs of toxicity in male and female animals. The treatment, until sacrifice time, did not produce any adverse reactions or any significant changes in the body weights (data not shown) as compared to untreated controls. In a similar work realized by Diaz et al. [11] the authors did not observe increases in the micronucleated erythrocytes frequency in mouse treated orally with extracts of this plant at doses of 500, 1000, and 2000 mg/kg body weight. Another study of toxicity performed with chloroform and hexane soluble fractions of* X. strumarium* reveals that the administration of a very high dose (5 g/kg body weight) for acute toxicity determination could not make any abnormal changes at gross as well as histopathological levels in the treated animals [28].

In our case, observed genotoxic effects were not confirmed* in vivo* with micronucleus test. This experiment evidenced our postulation indicating that nontoxic doses showed neither DNA damage nor toxic effects. Besides the usual arguments invoked to explain the* in vivo* negative performance of* in vitro *genotoxins, which deal mainly with the pharmacokinetic profile of absorption-distribution-metabolism-excretion, one may wonder if genotoxic response to* X. strumarium* extract was related to the reported cytotoxicity, which could implicate an indirect interference with nuclear functions to arrive to cell death, as it happens for many anticancer agents [24, 25, 31, 32].

We suggest that further examination of the cytotoxic potential of these compounds should be pursued. Further studies have to be performed using various cancer models to elucidate the exact mechanism by which* X. strumarium* extract exerts its cytotoxic action. In addition, new molecular studies are needed in order to elucidate the interaction of this compound in cell biology and the consequences for human health.

## 5. Conclusions

The data presented here constitute the most complete study on the genotoxic potential the aerial parts of* X. strumarium *L. and show that the extract can induce* in vitro *DNA damage at cytotoxic concentrations and, however, does not induce micronucleus in bone marrow of mice treated orally with extracts of this plant at dose of 500–2000 mg/kg body weight in our experimental conditions.

## Figures and Tables

**Table 1:** Mutagenicity testing of *Xanthium strumarium* extract in the *Salmonella*/microsome assay (TA 1535 and TA 1537).

Concentration (*μ*g/placa)	TA 1535 Mean revertants/plate ± SD		TA 1537 Mean revertants/plate ± SD	
	−S9	+S9	−S9	+S9
0^a^	26.00 ± 8.54	10.3 ± 2.52	9.67 ± 2.89	8.00 ± 0.0
50	21.67 ± 4.04	9.00 ± 1.73	9.33 ± 0.58	7.33 ± 2.08
150	23.67 ± 2.08	8.67 ± 4.16	8.00 ± 1.00	6.00 ± 2.00
500	28.33 ± 9.07	11.33 ± 2.52	14.00 ± 3.46	8.33 ± 3.21
1500	32.67 ± 2.08	10.33 ± 6.11	12.33 ± 3.08	6.67 ± 2.31
5000	28.67 ± 1.53	5.67 ± 2.52	16.3 ± 4.93	7.33 ± 4.04
NaA1.5^b^	405.3 ± 70.5∗∗			
CP 500^b^		228.3 ± 36.2∗∗		
9AA 100^b^			460.0 ± 50.0∗∗	
2AF 20^b^				580.0 ± 34.6∗∗

+S9: with liver microsomal S9 fraction; −S9: without liver microsomal S9 fraction.

^a^Negative control, dimethyl sulfoxide (DMSO).

^b^Positive controls. NaA: sodium azide; CP: cyclophosphamide; 9AA: 9-aminoacridine; 2AF: 2-aminofluorene.

∗∗ *P* < 0.01 (Student's *t*-test).

**Table 2:** Mutagenicity testing of *Xanthium strumarium* extract in the *Salmonella*/microsome assay (TA 100 and TA 98).

Concentration (*μ*g/placa)	TA 100 Mean revertants/plate ± SD		TA 98 Mean revertants/plate ± SD	
	−S9	+S9	−S9	+S9
0^a^	58.33 ± 16.56	62.60 ± 15.1	25.67 ± 3.79	38.33 ± 2.52
50	54.33 ± 5.13	64.10 ± 10.5	26.00 ± 4.36	38.67 ± 11.85
150	45.33 ± 4.73	60.04 ± 13.7	28.67 ± 7.64	45.67 ± 8.33
500	44.00 ± 2.00	60.33 ± 5.77	22.00 ± 5.29	35.67 ± 20.11
1500	46.67 ± 2.52	70.70 ± 7.37	26.00 ± 4.36	40.67 ± 8.62
5000	51.67 ± 3.21	67.31 ± 2.52	27.00 ± 2.65	38.33 ± 2.52
NaA1.5^b^	480.0 ± 22.6∗∗			
2AF 20^b^		2280.0 ± 169.7∗∗		3300.0 ± 141.4∗∗
PA 100^b^			992.0 ± 11.3∗∗	

+S9: with hepatic fraction S9; −S9: without hepatic fraction S9.

^a^Negative control, dimethyl sulfoxide (DMSO).

^b^Positive controls. NaA: sodium azide; 2AF: 2-aminofluorene; PA: pichrolonic acid.

**P < 0.01 (Student's *t*-test).

**Table 3:** Evaluation of chromosomal aberrations and mitotic index in CHO cells treated with whole extract of *Xanthium  strumarium* L.

Concentration (*μ*g/mL)	Number of cells	Aberrant cells^a^ (%)	Mitotic index (%)	Chromatid breaks	Chromosome breaks	^ b^Exch.	Total Ab.
DMSO 1%	150	1.33	74	0.66	0.66		1.33

3 h + 15 h recovery							
10	150	1.33	77	0.66	0.66		1.33
25	150	4.00∗	62	0.66	3.33		4.00
45	150	8.66∗∗	32	2.00	7.33		9.33

18 h continuous							
10	150	2.00	44	0.66	1.33		2.00
25	150	4.00∗	53	2.66	2.66		5.33
45	150	NM	2				
Mit C^c^	100	23.00∗∗		21.00		12.00	33.00

RX^d^	100	38.00∗∗		2.00	48.00	16.00	66.00

^a^Excluding gaps; NM: no mitoses; ^b^chromosome and chromatid exchanges; ^c^mitomycin C positive control (0.05 *μ*g/mL); ^d^RX: positive control (2 Gy); ∗*P* < 0.05; ∗∗*P* < 0.01 versus DMSO sample.

**Table 4:** Micronucleus test results in mice treated with whole extract of *Xanthium strumarium* L.

Dose (mg/kg)	Female		Male	
	PCE/NCE^a^ ± SD	MNPCE/2000^b^ ± SD	PCE/NCE^a^ ± SD	MNPCE/2000^b^ ± SD
0	0.46 ± 1.32	0.18 ± 0.43	0.35 ± 1.39	0.20 ± 0.24
500	0.07 ± 1.57	0.03 ± 0.08	0.10 ± 1.70	0.03 ± 0.11
1000	0.28 ± 1.58	0.06 ± 0.08	0.16 ± 1.51	0.03 ± 0.07
2000	0.08 ± 1.46	0.03 ± 0.11	0.07 ± 1.46	0.03 ± 0.11
Ethanol^c^	0.05 ± 1.39	0.01 ± 0.08	0.32 ± 1.45	0.04 ± 0.08
CP^d^	0.06 ± 0.63∗∗	1.80 ± 7.62∗∗	0.07 ± 0.53∗∗	0.75 ± 5.38∗∗

^a^PCE/NCE: polychromatic (PCE) to normochromatic (NCE) erythrocyte ratio; ^b^MNPCE/2000: frequency of micronucleated PCE; ^c^Negative control: ethanol 60%; ^d^Positive control: cyclophosphamide 20 mg/Kg. ∗∗*P* < 0.01 (Student's *t*-test).
